# Metal–Phenolic Film Coated Quartz Crystal Microbalance as a Selective Sensor for Methanol Detection in Alcoholic Beverages

**DOI:** 10.3390/mi14061274

**Published:** 2023-06-20

**Authors:** Karekin D. Esmeryan, Yuliyan Lazarov, Teodor Grakov, Yulian I. Fedchenko, Lazar G. Vergov, Stefan Staykov

**Affiliations:** Acoustoelectronics Laboratory, Georgi Nadjakov Institute of Solid State Physics, Bulgarian Academy of Sciences, 72, Tzarigradsko Chaussee Blvd., 1784 Sofia, Bulgaria

**Keywords:** hydrophobicity, metal–phenolic film, methanol detection, quartz crystal microbalance, selectivity

## Abstract

The facile real-time monitoring of methyl content in fermented beverages is of fundamental significance in the alcohol and restaurant industry, since as little as 4 mL of methanol entering the blood may cause intoxication or blindness. So far, the practical applicability of available methanol sensors, including the piezoresonance analogs, is somewhat limited to laboratory use due to the complexity and bulkiness of the measuring equipment involving multistep procedures. This article introduces a hydrophobic metal–phenolic film-coated quartz crystal microbalance (MPF-QCM) as a novel streamlined detector of methanol in alcoholic drinks. Unlike other QCM-based alcohol sensors, our device operates under saturated vapor pressure conditions, permitting rapid detection of methyl fractions up to seven times below the tolerable levels in spirits (e.g., whisky) while effectively suppressing the cross-sensitivity to interfering chemical compounds such as water, petroleum ether or ammonium hydroxide. Furthermore, the good surface adhesion of metal–phenolic complexes endows the MPF-QCM with superior long-term stability, contributing to the repeatable and reversible physical sorption of the target analytes. These features, combined with the lack of mass flow controllers, valves and connecting pipes delivering the gas mixture, outline the likelihood for future design of a portable MPF-QCM prototype suitable to point-of-use analysis in drinking establishments.

## 1. Introduction

Alcohol plays a central role in the festive and social events worldwide, and the first traces of beer and fermented beverages date back to 11,000–1200 B.C. [1,2]. Beyond its recreational and ritual functions, alcohol is an integral part of the modern medicine, agriculture, petroleum and cosmetic industry, being used for the manufacturing of medicinal liquids, liniments, perfumes, drugs, aftershaves, automobile fuels, plastics, etc. [2,3,4,5]. Nevertheless, some alcohol fractions are highly hazardous to human health and the unintentional ingestion, inhalation or skin absorption of methanol, for instance, may lead to irreversible tissue damage, blindness or even lethal end [6,7]. Therefore, inventing cutting-edge approaches for monitoring the air quality in distillation premises or detecting the presence of methanol in spirits is a task of direct relevance to public healthcare [8].

Gas chromatography is one of the well-known techniques for ascertaining the percentage of methyl alcohol in fermented beverages [9]. In essence, the liquid target analyte is placed in a gas chromatograph and upon evaporation at temperatures above the boiling point, the gaseous molecules are mixed with a carrier gas and delivered to separation columns equipped with a detector. Undoubtedly, the gas chromatographs are accurate, but also cost-ineffective and require trained personnel to avoid analyte stuck in the columns or gas leaks, which hinders the widespread use of these machines in low-income countries [10].

The tremendous progress of engineering sciences allows the launch of innovative methods for methanol screening in alcoholic products [11,12,13,14,15,16,17,18,19,20,21]. Some of them rely on enzymatic, colorimetric and spectroscopic quantification [11,12,13], chiral nematic liquid crystals [14], handheld metal oxide microsensors [7], resistive sensors [15,16,17], metal–organic frameworks [18], immunosensors [19] or optical and microwave-based sensors [20,21]. Despite the valuable contribution of each of the mentioned strategies, a few shortcomings would impede their practical implementability. First, the sensitivity of resistive detectors is temperature dependent and optimal performance is unattainable below 75 °C [17], which is far above the standard room temperature conditions. Second, the optical elements need complex equipment to generate the specific wavelength of light [20], while the metal–organic frameworks are highly influenced by the ambient humidity [18]. Third, the metal oxide sensors and nematic liquid crystals are excellent examples of small-scale portable devices [7,14]. However, the slow recovery of the detector’s baseline [7] and the color/light intensity-related sensor signal [14] limit the efficiency of these apparatuses or make them unusable by people with color blindness. Finally, the inclusion of spectroscopy measurements and different chemical or biological agents [11,12,13,19] implies cost ineffectiveness and applicability of the particular assays mostly to laboratory conditions.

Another opportunity for highly sensitive detection of methanol in liquors emanates from the advantages of quartz crystal microbalances (QCMs)—miniature piezoelectric disk-shaped quartz crystals mounted between two metal electrodes [22]. In cases of alcohol analysis, the resonance frequency of these devices alters proportionally to the mass and type of organic molecules adhering to the sensor surface [22]. If the latter is appropriately modified with a thin and selective functional coating, the QCMs can operate in a wide dynamic range (the span of analyte concentrations inducing detectable differences in the resonance frequency) and provide absolute sensitivity of ng/cm^2^, low detection limit (ppb) and fast response–recovery time (within seconds), unattainable with the other available techniques [23,24,25,26,27,28,29,30,31,32,33,34]. Regrettably, many QCMs exhibit commensurable to the useful signal cross-sensitivity to ambient humidity (see Figures 3 and 4 in Ref. [27]), so this shortcoming may require a combination of piezoresonance sensor arrays and pattern recognition approaches [23,32,35]. Alternatively, the hydrophobic modification of the quartz crystal’s upper electrode area is an artful measure against the interfering water vapor molecules due to the suppressed surface sorption of moisture and increased nucleation-free energy barrier (ceasing the formation of water condensates occupying the active sites intended to interact with the alcohol) [36,37,38,39,40,41]. Nonetheless, the ordinary experimental setup for organic solvents detection encompasses multiple mass flow controllers, valves and pipes [26], making such equipment cumbersome for out-of-lab applications. In addition, the target analyte is often injected in the test chamber via a syringe and a ventilator is used to distribute the alcohol vapors uniformly [24,25,26,27,28,29,30,31]. This could instigate measurement uncertainties, because the liquid–vapor–liquid phase transitions are stochastic [42] and any spontaneous nucleation may influence the oscillation frequency due to unpredictable alterations in the gas concentration. The existing disadvantages outlined above indicate that the contemporary QCM-based methanol sensing platforms are suitable predominantly to track the levels of toxic volatile compounds indoors (e.g., in distillation premises), so further study is mandatory to propose and develop a QCM configuration ensuring portability and adaptability to drinking establishments.

Within this article, we advance the current state of the art by suggesting a hydrophobic metal–phenolic film-coated QCM possessing the desirable capabilities of a point-of-use device for discrimination of organic solvents. We reveal experimentally that the water-repellent interface blocks the cross-sensitivity to different interfering chemical compounds, enabling selective detection of methanol in fermented beverages with a resolution of 2.7 µL/mL. Another major benefit of our sensor system is the inherently high adhesion of metal–phenolic coatings [43], hampering the interfacial delamination and providing excellent long-term stability and reversibility of the quartz resonator even after 50 cycles of vapor sorption–desorption. Meanwhile, maintaining saturated vapor pressure in the test chamber eliminates the use of flow meters, valves and connection pipes, which is a prerequisite for possible incorporation of the measuring setup in a standalone instrument with potential for future utilization in nightclubs or discotheques.

## 2. Materials and Methods

### 2.1. Materials

Ethanol (96 wt%), methanol (99.5 wt%), isopropyl alcohol (99.8 wt%), ammonium hydroxide (25 wt%), petroleum ether (CAS Number 8032-32-4), iron (III) chloride hexahydrate (FeCl_3_·6H_2_O) and phenol (99 wt.% C_6_H_6_O) were supplied by Valerus Ltd. (Sofia, Bulgaria). Gold electrode AT-cut quartz resonators with diameter of 25.4 mm and 5 MHz resonance frequency were purchased from Stanford Research Systems (Sunnyvale, CA, USA).

### 2.2. Synthesis and Deposition of the Metal–Phenolic Film

The metal–phenolic film was deposited by adopting specific technical protocols reported in the scientific literature [43,44]. Accounting for the water solubility of chosen compounds [45,46], 8.3 g phenol and 91.2 g FeCl_3_·6H_2_O were dissolved in two beakers filled with 100 mL distilled water and then mixed together in 1:1 volume ratio. A 5 MHz QCM was fixed in a spin coater and after 2 h sedimentation of the metal–phenolic suspension, 0.3 mL of the heavy fraction (precipitate) were deposited on the resonator’s active area via a pipette. Spinning at 500 rpm for 20 s and 1800 rpm for 120 s, followed by 48 h drying at room temperature (*T* ~ 20 ± 2 °C; *RH* ~ 50 ± 10 %), led to the formation of a uniform metal–organic functional coating. Direct immersion of the QCM in a metal–phenolic solution is not preferable, because it is still unclear how the bottom electrode is protected from unwanted film deposition [44], which might cease or damp the oscillations. Choosing iron (III) chloride hexahydrate (FeCl_3_·6H_2_O) as a sensing material, instead of other metallic salts such as aluminum chloride hexahydrate, for example, is justified by the fact that the assembly of iron–polyphenol complexes can be accurately controlled and adjusted via different methods, resulting in a coating with custom physicochemical features including permeability, stiffness, degradability and wettability [43,44,47]. This opens a lot of possibilities for tuning the QCM’s selectivity and sensitivity depending on the practical application.

### 2.3. Materials Characterization

Surface characterization of the metal–phenolic film was accomplished via scanning electron microscopy (SEM), energy dispersive spectroscopy (EDS), X-ray photoelectron spectroscopy (XPS) and wettability analysis. Top-view SEM images were obtained through a JEOL JSM-5510 scanning electron microscope (JEOL, Tokyo, Japan) at magnifications of 1–5 kX. Elemental analysis was executed with an energy dispersive spectrometer Bruker at 126 keV with an EDAX detector having 1.3 mm^2^ active area. The standard deviation values of detected chemical elements were automatically generated by the EDS instrumentation. X-ray photoelectron lines were recorded via an Axis Supra electron spectrometer using achromatic AlKα radiation at 1486.6 eV. The photoelectron core levels of chemical elements were processed and corrected by subtracting a Shirley-type background, and quantified by the peak area and Scofield’s photoionization cross-sections. The chemical states were identified by deconvoluting the high-resolution spectra with XPSPEAK41 software. To define the surface wettability, static contact angle measurements were performed via an optical measurement system OCA 15EC (DataPhysics, Stuttgart, Germany) by dispensing 10 μL distilled water droplets on three surface areas.

### 2.4. Experimental Section

**Figure 1:**
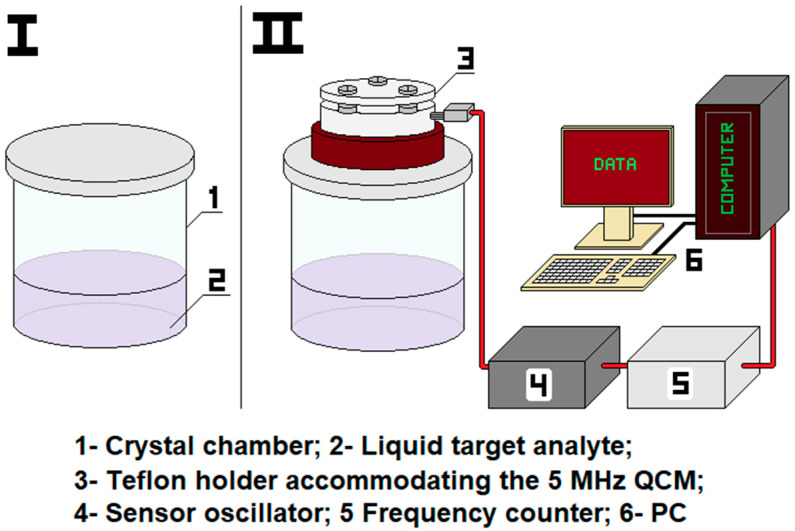
Scheme of the experimental setup.

The proposed system for alcohol quality assessment is composed of a 500 mL crystal chamber with removable sheet-iron lid, a 5 MHz metal–phenolic film-coated QCM (MPF-QCM) placed in a Teflon holder firmly attached to a second sheet-iron lid, a Maxtek sensor oscillator, a frequency counter HM 8123 and a personal computer (PC), as illustrated in Figure 1. Initially, 100 mL of a given target analyte (here, water, ammonium hydroxide, petroleum ether, ethanol, methanol and isopropanol) were poured into the chamber and left for 10 min until liquid–vapor thermodynamic equilibrium was reached (saturated vapor pressure), ensuring the presence of 100% pure target gas in the surroundings above the liquid. Simultaneously, the QCM was gently fixed in the holder, electrically connected to the oscillator and frequency counter, and left to stabilize its resonance frequency (∆*f_air_* ≤ ±1 Hz/s).

Once this happened and the entire system was equilibrated (transient responses around ±1 Hz/s, indicated on the frequency counter’s display), the active area of the quartz crystal resonator was embedded in the chamber and exposed to the analyte’s saturated vapor. Subsequently, the evolution of sensor signal was recorded on a PC at 1 s gate time via LabView 6 software until the observed frequency shifts diminished to ±1 Hz/s (fell in the short-term stability range). All assays were implemented in three independent measurement cycles at equal conditions (*T_air_* = *T_liquid_* ~ 20 ± 2 °C; *RH_air_* ~ 50 ± 10%), since this is a standard in QCM technologies [25,29,33,39], ensuring invariable vapor pressure *P* of each analyte and constant saturated vapor concentrations from experiment-to-experiment. Importantly, maintaining unaltered ambient humidity values outside the test chamber (*RH_air_* ~ 50 ± 10%) was feasible via the built-in air conditioning system of “Acoustoelectronics” Laboratory at ISSP-BAS. It must be noted that the frequency shifts triggered by the gas phases of selected analytes in air were not measured, since this contradicts the main concept of our research, which is to promote a sensor platform that does not rely on flow meters, valves, pipes, etc. Instead, the possible adverse effects of relative humidity on the MPF-QCM’s sensitivity were evaluated by studying its resonance response to saturated water vapor (RH = 100%) at room temperature (*T_air_* = *T_water_* ~ 20 ± 2 °C), achieved by filling the crystal chamber (see Figure 1) with 100 mL distilled water and waiting for thermodynamic equilibration of the closed system, corresponding to 100% water molecules occupying the empty space above the liquid.

As a proof of concept, the experimental procedures were repeated by subjecting the MPF-QCM to the vapors of 100 mL Dewar’s whisky with gradually increasing methanol or isopropanol content. Knowing that an oral intake of ~3.16–11.85 g (~4–15 mL) pure methanol inflicts blindness [48] and considering the tolerable concentrations of methanol in 100 mL alcoholic drinks (2 vol.% = 2 mL) [49], we consequently inserted in the spirit 4–9.5–15 mL of methanol or isopropanol. The latter was selected as a control with the aim of comparing the emerging frequency shifts and facilitating the description of sensing mechanisms. In order to verify that the sensor’s selectivity is due to the metal–phenolic functionalization rather than a measurement artifact, an uncoated resonator was exposed to the saturated vapors of distilled water and methanol.

### 2.5. Defining the Saturated Vapor Concentrations

The saturated vapor concentrations *C* (ppm) of pure target analytes were calculated based on the ideal gas law [39]. Since the state of a particular gas depends on the temperature, pressure and volume,

(1) $$\frac{P}{RT}=\frac{n}{V}$$

where *P*—vapor pressure of liquid target analyte (Pa); *T*—its temperature (K); *R*—universal gas constant (J/mol × K); *n*—the quantity of gas/vapor (mol); and *V*—volume of the gas/vapor (L). The number of gas molecules in a certain volume yields the concentration in mol/L and taking into account the molecular mass of each analyte, the dimension of *C* is converted to ppm.

In case of a mixture of liquids, as upon detection of toxic methanol or isopropanol in whisky (see Section 2.4), the partial vapor pressure of different alcohol fractions *P_i_* is related to the equilibrium vapor pressure of single compounds multiplied by their mole fraction *x_i_*, according to Raoult’s law for ideal liquid mixtures:

(2) $$P_{i}=Px_{i}$$

In a two-component mixture such as whisky–methanol or whisky–isopropanol, the mole fractions of whisky, methanol and isopropanol can be found as follows:

(3) $$x_{i}=\frac{n_{i}}{n_{tot}}$$

where *n_i_*—the moles of first fraction (e.g., methyl or isopropyl) and *n_tot_*—the total number of moles in the solution (comprising both whisky–methyl or whisky–isopropyl). Substituting the values of *P_i_* for whisky, methanol and isopropanol in Equation (1) yields their concentration in the ambiance above the alcohol mixture in ppm. To simplify the calculations, and since Dewar’s whisky is a complex mixture of alcohol, water and some other ingredients, its mole fraction is approximated to that of ethanol (96 wt%).

## 3. Results

### 3.1. Physicochemical Profile of the Metal–Phenolic Film

**Figure 2:**
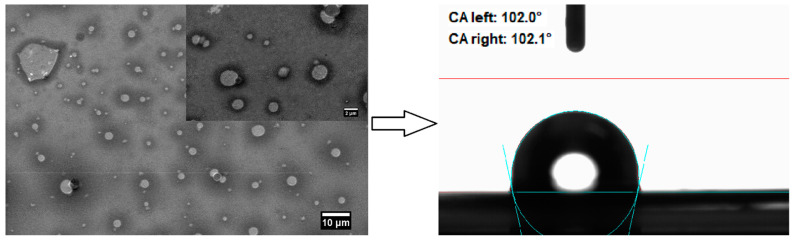
Morphology, structure and surface wettability of the metal–phenolic film.

Figure 2 shows the morphostructure and surface wettability of the metal–phenolic film.

As seen, the metal–ligand complexes existing in the main suspension lead to the deposition of homogeneous coating consisting of longitudinal cross-linked molecules and residual FeCl_3_ solid particles with varying sizes [44,47]. Phenol possesses one hydroxyl group connected to an aromatic phenyl ring, triggering partial transfer of negative charges from oxygen to the ring, delocalization of the charges and ability for hydrogen bonding with water molecules [45]. Hence, the coating’s hydrophobicity (water contact angle of 102°) is attributed to the attachment of “electronwithdrawing” group (e.g., -F or -Cl) to the phenyl during the metal–phenol molecular interactions, affecting the ring’s electron density and the dipole moment and acidity of the molecule [45].

**Figure 3:**
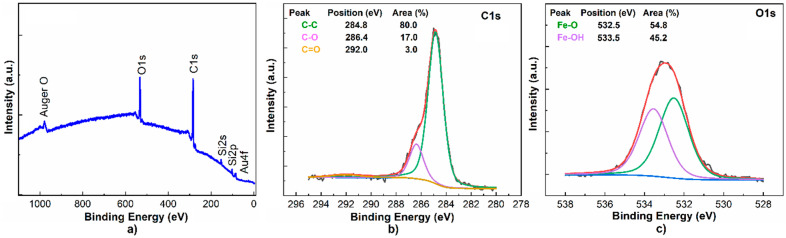
XPS (**a**) scan survey, (**b**) C1s and (**c**) O1s photoelectron lines of MPF-QCM.

Elucidating the elemental composition and surface chemical state of the metal–phenolic coating is feasible via EDS and XPS analysis, as summarized in Table 1 and Figure 3.

The probing of the QCM’s surface reveals six primary chemical elements, including carbon, oxygen, silicon, gold, iron and chlorine, associated with the quartz crystal–gold electrode structure of the microbalance and hydrocarbon–iron chloride species due to the metal–phenolic treatment. Interestingly, the atomic percentage of Fe and Cl in the area without FeCl_3_ particles (see Table 1) correlates to that disclosed in ref. [47], but these elements are not registered by the XPS. Perhaps its short beam penetration depth blocks the access to Fe atoms, longitudinally arranged between underlying and overlying phenol molecules [44]. Nonetheless, the C-C and C-O peaks in the C1s spectrum, along with the Fe-O and F-OH functionalities appearing at ~532.5 eV and ~533.5 eV in the O1s core level, confirm the deposition of a metal–phenolic coating. Fe-O bonding occurs by coordination among phenol and iron, whereas Fe-OH groups are ascribed to hydrogen bonding with water molecules [44,47].

### 3.2. Detection of Organic Solvents and Interfering Chemical Compounds

**Figure 4:**
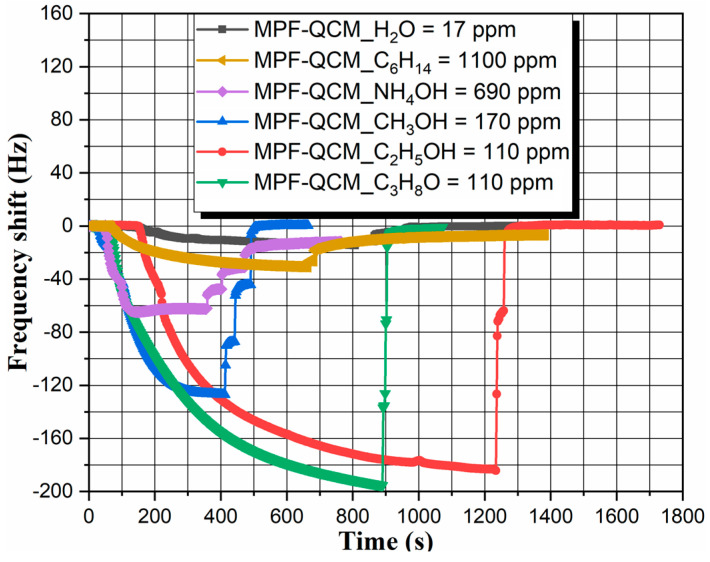
Resonance response of an MPF-QCM induced by the saturated vapor of water, ammonium hydroxide, petroleum ether, methanol, ethanol and isopropanol at *T_air_* = *T_liquid_* ~ 20 ± 2 °C. Each curve is an average of three measurement cycles. The gas concentrations of all target analytes are obtained by using the corresponding saturated vapor pressure values at *T_air_* = *T_liquid_* ~ 20 ± 2 °C (available in ref. [46] or other handbooks of physics and chemistry) and substituting them in Equation (1).

The first part of experiments concerns the sensor performance of MPF-QCM towards different chemical compounds and organic solvents under saturated vapor pressure conditions, as illustrated in Figure 4. Its brief overview reveals ~2–13 times lower frequency response ∆*f* of the MPF-QCM to water, ammonium hydroxide (NH_4_OH) and petroleum ether (C_6_H_14_) molecules compared to the alcohol vapors. Furthermore, the response time *t_res_*, defined as the time range where ∆*f* > ±1 Hz, differs significantly from analyte-to-analyte and the slowest sorption–desorption equilibrium is gained by ethanol. In addition, the microbalance restores its resonance frequency within seconds (up to 191 s) after removal from the test chamber, except in the cases of NH_4_OH and C_6_H_14_ sensing, where the regeneration takes about 5–6 h—a phenomenon that will be discussed later in the article. Beyond the unusually slow desorption of NH_4_OH and C_6_H_14_ molecules, our MPF-QCM indicates ~4–18 times faster baseline recovery juxtaposed to the handheld metal oxide sensors (~5–27 min to methanol and ethanol) [7] and better selectivity than other QCM detectors (see Figure 11a in ref. [31]). It is worth mentioning that the rapid desorption of vapor molecules at room temperature is related to the lack of irreversible (permanent) chemical bonding between the adsorbent (i.e., the metal–phenolic complexes) and adsorbate.

**Figure 5:**
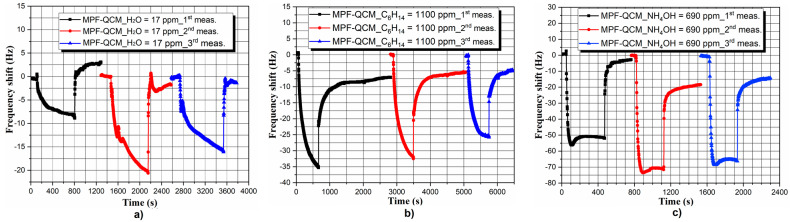
Repeatability and reversibility of the MPF-QCM’s response to saturated (**a**) water, (**b**) ammonium hydroxide and (**c**) petroleum ether vapors at *T_air_* = *T_liquid_* ~ 20 ± 2 °C.

**Figure 6:**
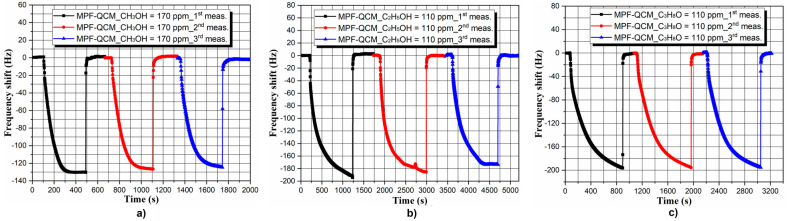
Repeatability and reversibility of the MPF-QCM’s response to saturated (**a**) methanol, (**b**) ethanol and (**c**) isopropanol vapors at *T_air_* = *T_liquid_* ~ 20 ± 2 °C.

Defining the repeatability and reversibility of the signal is an inevitable step prior to the incorporation of MPF-QCM in portable systems for methanol detection in fermented beverages. As demonstrated in Figure 5 and Figure 6, the sensor response of the as-prepared QCM is repeatable for all analytes with maximum frequency deviations within ±9 Hz, identified during ethanol and NH_4_OH sorption.

Principally, the slight inconsistency of the signal during the probing of interfering chemical fractions such as water, petroleum ether and ammonium hydroxide (see Figure 5) could be considered as unsatisfactory QCM performance, but ±9 Hz variations in the average frequency response are something quite common (see Figure 9 in refs. [29,30]) and mainly attributed to the unavoidable ambient temperature and humidity fluctuations (±2 °C; ±10%, respectively). Nevertheless, no significant baseline drifts are observed throughout the assays, meaning that the molecular interactions are mainly physical (no chemical modifications of the adsorbent’s surface [50]) and confirming the excellent long-term stability of the MPF-QCM configuration.

**Figure 7:**
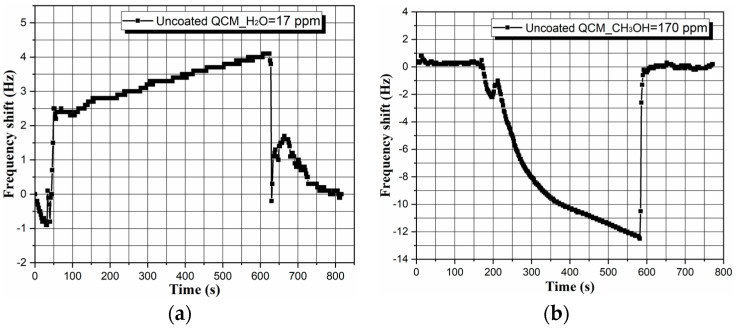
Sensor response of an uncoated 5 MHz QCM towards saturated (**a**) water and (**b**) methanol vapors at *T_air_* = *T_liquid_* ~ 20 ± 2 °C.

It could be argued that the sensor’s sensitivity and selectivity are not directly related to the chemically functionalized active area of the microbalance, so we tested the sorption capacity of an uncoated 5 MHz QCM, revealed in Figure 7. The absence of a chemical interface inhibits the immobilization of a finite mass of water and methanol vapors, and the resultant perturbations in the frequency of uncoated QCM are negligible (∆*f_H2O_* ~+4 Hz; ∆*f_CH3OH_* ~−12 Hz), reaffirming the role of metal–phenolic film in the detection process.

### 3.3. Sorption–Desorption Kinetics and Sensing Mechanisms

Of scientific interest are the observed distinctions in the sorption–desorption kinetics of target analytes that would help to elucidate the detection mechanisms if scrutinizing the experimental data collected in Table 2.

As reported elsewhere, the sorption–desorption velocity depends on the number of gas molecules and at low concentrations, the sorption should be much faster than the desorption [26]. From that point of view, the slower rate of water, petroleum ether, ethanol and isopropanol sorption collated to methanol or ammonium hydroxide is surprising, but it has its explanation. For example, although the low volatility of water (*P* ~2.34 kPa at *T* = 20 °C) sets low gas concentration *C* = 17 ppm, implying fast response time, the water vapor sorption is strongly delayed due to the hydrophobicity of the sensor surface. It weakens the solid–vapor attraction forces and increases the free energy barrier for heterogeneous nucleation [39,41], thus retarding the initial chemisorption (i.e., hydrogen bonding) and subsequent physisorption (i.e., the inception of condensed adsorbate layers). In addition, the chemical non-polarity of petroleum ether determines weak hydrophobic and/or van der Waals molecular attraction to the metal–phenolic film and hence, the physisorption takes longer time (see Table 1). In terms of the alcohols, methyl has the lowest gas-phase acidity compared to isopropyl and ethyl [51], so the higher electron donating affinity of methylene groups allows faster stabilization of the negatively charged iron hydroxide, changing its dipole moment and electron density [45], and leading to accelerated sorption—an effect observed also for soot-coated QCM-based alcohol sensors [39]. Therefore, despite the higher gas concentration of methanol above its liquid phase (see Table 2), the respective response time of the MPF-QCM is shorter compared to the case of ethanol and isopropanol sensing. In addition, the sensor signal induced by the alcohols is proportional to their molecular weight, in compliance with previous research [27,28,39]. The existence of electrophile (“electron-withdrawing”) species, such as FeCl_3_ particles, on the metal–phenolic-coated active area makes the sorption reversible [45].

Referring to the reversibility, the unusually long recovery time upon desorption of NH_4_OH and C_6_H_14_ could be justified by the molecular size of chemical compounds. Ammonium hydroxide and petroleum ether molecules are smaller than the alcohol counterparts (NH_4_OH/C_6_H_14_—0.26–0.33 nm; CH_3_OH/C_2_H_5_OH/C_3_H_8_O—0.38–0.49 nm) and probably, they diffuse into the porous structure of the adsorbent, resulting in stronger adhesion of the condensed monolayers and more difficult desorption [45]. It can be summarized that the MPF-QCM successfully suppresses the undesired effects of interfering analytes and shows good alcohol discrimination behavior primarily due to the hydrophobic character of metal–phenolic complexes. They hinder the hydrogen bonding with hydroxyl functional groups of H_2_O and NH_4_OH, and set weak molecular interactions with C_6_H_14_. Meanwhile, the as-prepared sensor differentiates the distinct alcohol vapors based on their acidity and molecular weight—parameters regulating the sorption–desorption kinetics.

### 3.4. Detection of Methanol and Isopropanol in Alcoholic Drinks

**Figure 8:**
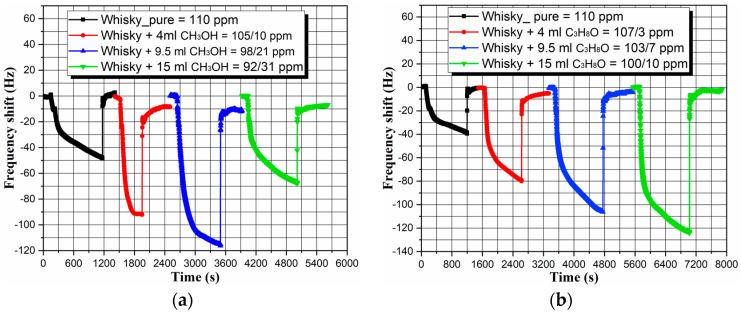
Sensor response of the MPF-QCM to gradually increasing content of (**a**) methanol and (**b**) isopropanol in 100 mL Dewar’s whisky.

Considering the good selectivity, repeatability and reversibility of the MPF-QCM, its efficiency as a detector of methyl and isopropyl alcohol in spirits is studied and presented in Figure 8. The initially low vol.% of methyl in the whisky triggers frequency downshifts, but eventually, the cumulative methanol content leads to frequency upshifts (see Figure 8a), because methanol is lighter than ethanol. Resultantly, the larger the methyl fraction in the saturated vapor mixture, the smaller the total molecular mass. In other words, the continuous replacement of heavier gas molecules with lighter ones displaces the resonance frequency upwards, in perfect agreement with the operation principles of the QCM [22].

To be explained clearer, let’s assume that the weight of two molecules, *X* and *Y*, is *W_X_* = 1 ng and *W_Y_* = 0.5 ng, accordingly. Placing hundred *X* molecules on an analytical balance (just as an example) yields mass *W_X_* = 100 ng. Now, if thirty *Y* molecules are added to the balance, the overall mass will change as follows: *W_XY_*_1_ = *W_X_*_100_ + *W_Y_*_30_ = 100 + 15 = 115 ng. The addition of another thirty *Y* molecules will change the mass to *W_XY_*_2_ = *W_X_*_100_ + *W_Y_*_60_ = 100 + 30 = 130 ng. However, if, at some point, fifty of the attached *X* molecules are replaced by fifty *Y* molecules, the total mass will decrease to *W_XY_*_3_ = *W_X_*_50_ + *W_Y_*_110_ = 50 + 55 = 105 ng. That said, the initial insertion of small amounts of methanol in the whisky (e.g., 4 mL/100 mL) does not affect the reactivity of the vapor phase, because most of it is composed of ethanol molecules predominantly occupying the active sites of the metal–phenolic film, so the mass loading increases and the resonance frequency decreases. This process continues until a threshold methanol concentration is reached (herein, 15 mL/100 mL), above which the more volatile and chemically reactive methanol molecules start preferentially attaching to the surface sites at the expense of ethanol counterparts. Then, instead of increasing, the total mass decreases (as exemplified supra), automatically shifting the resonance frequency upwards, which is exactly what we observe. Our hypothesis is unambiguously proven by inserting the same vol.% of isopropanol (heavier molecules) in the whisky, which engenders linear downward movement of the sensor response due to the progressively increasing molecular mass (see Figure 8b).

Since ∆*f_whisky_* − ∆*f_whisky_4mLCH3OH_* = 45 Hz, the sensitivity of the MPF-QCM to the availability of 4 mL methanol in 100 mL whisky (the minimum concentration causing blindness [48]) is ∆*f*/∆*C* = 45/4 = 11.25 Hz/mL. Considering a short-term frequency stability of ±1 Hz/s and selecting a signal-to-noise ratio of 3:1 [50] yields maximum sensor resolution (limit of detection) *LoD* = 0.27 mL/s or 2.7 µL/mL. This means that our MPF-QCM enables the registration of methyl fractions up to seven times below their maximum tolerable concentration in alcoholic drinks [49]. On the other hand, if the as-prepared sensor is intended to detect target organic vapors in the absence of a liquid phase (for instance, to facilitate the air quality control in distillation premises), the frequency shifts of 45 Hz to 10 ppm methanol imply a detection limit of 666 ppb/s, which is commensurable to the performance of other QCM-based alcohol sensors [24,25,28]. Finally, the resonance response to pure whisky is repeatable in both measurements (∆*f* = 45 ± 5 Hz, as seen in Figure 8), warranting accurate device calibration prior to the assays.

### 3.5. Our Vision for Designing a Portable MPF-QCM-Based Sensor System

The next few paragraphs discuss the design of an MPF-QCM-based handheld sensor system, potentially applicable to restaurants, gambling venues, nightclubs, etc. All of these places serve alcohol on a regular basis and the monitoring of its quality (both by owners and customers) may be accomplished via a simple portable device. In our perspective, it should be composed of a miniature (cm-sized) metal corps accommodating the QCM module (e.g., a 5 MHz MPF-QCM mounted in a Teflon holder), electrically connected to a built-in sensor oscillator and a frequency counter, a digital display, a built-in power source and an on/off switch. According to one exemplary embodiment, 10 mL of a given beverage are injected into a 20 mL plastic or glass container with a removable lid, whose diameter matches that of the QCM module. After 2–3 min, the lid is moved aside and the module is firmly attached to the container by means of a lever. The real-time frequency shifts are shown on the digital display along with the elapsed time. Hence, the possible presence of methanol in the drinks will be ascertained in-situ by comparing the emerging signal (both frequency shifts and response time) with pre-determined calibration curves for each liquor (e.g., vodka, whisky, grape brandy, vermouth, cognac, tequila, gin, etc.). Our approach might be contested with the argumentation that the QCM must be thermostated and that any thermal drifts could also change the saturated vapor pressure of alcohols. While this is logical, it should be noted that the aforementioned premises operate under standard and constant room temperature conditions. Even if, by some reason, the interior temperature differs substantially from the norm (e.g., due to failure of the air conditioner), the resonance response could be equilibrated by recording the sensor signal of a set of alcohols in a wide temperature range. Moreover, identifying the dynamic sensing behavior of the MPF-QCM to different concentrations of methanol and/or ethanol–methanol mixtures with larger volume proportions is mandatory, as well as examining the gas-sensing performances under variable ambient humidity conditions. Obviously, these tasks cannot be implemented in a straightforward manner, but they are planned as future research, hopefully as part of a funded project.

## 4. Conclusions

The spin coating deposition of a metal–phenolic suspension on the active area of a 5 MHz gold-electrode QCM converted the latter into hydrophobic with a water contact angle above 100°. In turn, the functionalized sensor surface successfully suppressed the adsorption of water, petroleum ether and ammonium hydroxide vapors, while facilitating the preferential attachment of organic solvent molecules with different alkyl chain lengths. The observed differences in the resonance frequency behavior and response time were found to depend on the gas-phase acidity, molecular weight and size of alcohol analytes, promoting highly sensitive detection of methyl fractions in whisky based on changes in the mass of saturated ethanol–methanol vapor mixture. In view of the excellent long-term stability, repeatability and reversibility of the MPF-QCM, our device has strong potential for integrability in compact and handy sensor systems used to assess the quality of alcoholic drinks.

## 5. Patents

A Bulgarian patent application (No. 113673) was submitted on 8 March 2023 as a result of the work reported herein.

## Figures and Tables

**Table 1:** Elemental composition of MPF-QCM in the areas without and with iron chloride particles. The EDS graphic spectra are available as Figures S1 and S2 in Supplementary Materials.

Surface Area of the MPF-QCM	Chemical Element (at.%)					
	*C*	*O*	*Si*	*Au*	*Fe*	*Cl*
Without FeCl_3_ particles	20.5	44.7	26.4	7.5	0.7	0.2
With FeCl_3_ particles	5.5	50.4	3.2	2.6	30.3	8

**Table 2:** Maximum frequency shift ∆*f*, response time *t_res_* and recovery time *t_rec_* of the sensor caused by the saturated vapor concentrations *C* of selected liquid target analytes. The sign (±) denotes the standard deviation of the data for three experimental cycles.

Analyte	Molecular Mass *m* (g/mol)	*P* (kPa)	*C* (ppm)	*T_air_* = *T_liquid_* (°C)	∆*f* (Hz)	*t_res_* (s)	*t_rec_* (s)
H_2_O	18.2	2.34	17	20 ± 2	−15 ± 6	793 ± 79	191 ± 16
C_6_H_14_	82.2	31	1100	20 ± 2	−31 ± 4	609 ± 10	n/a
NH_4_OH	35.04	48	690	20 ± 2	−67 ± 9	71 ± 9	n/a
CH_3_OH	32.04	13.02	170	20 ± 2	−127 ± 2	329 ± 62	78 ± 8
C_2_H_5_OH	46.07	5.95	110	20 ± 2	−185 ± 9	1032 ± 66	90 ± 20
C_3_H_8_O	60.1	4.4	110	20 ± 2	−196 ± 1	837 ± 14	94 ± 15

## Data Availability

All data needed to evaluate the conclusions in this paper are presented duly. Supplementary data related to this research may be requested from the authors.

## Supplementary Materials

The following are available online at https://www.mdpi.com/article/10.3390/mi14061274/s1. Figure S1: EDX spectrum of the MPF-QCM in the areas without FeCl_3_ particles; Figure S2: EDX spectrum of the MPF-QCM in the areas with FeCl_3_ particles.
