# Supplementary material for: Metal–Phenolic Film Coated Quartz Crystal Microbalance as a Selective Sensor for Methanol Detection in Alcoholic Beverages
Source: Micromachines (Basel). 2023 Jun 20;14(6):1274. doi: 10.3390/mi14061274 (PMC10302850; doi:10.3390/mi14061274)
Supplement: Supplementary file 1 [file micromachines-14-01274-s001.zip › micromachines-2397423-supplementary.pdf]

micromachines

MDPI

Supplementary Materials

Metal–Phenolic Film-Coated Quartz Crystal Microbalance as a Selective Sensor for Methanol Detection in Alcoholic Beverages

Karekin D. Esmeryan *, Yuliyan Lazarov, Teodor Grakov, Yulian I. Fedchenko, Lazar G. Vergov and Stefan Staykov

Acoustoelectronics Laboratory, Georgi Nadjakov Institute of Solid State Physics, Bulgarian Academy of Sciences, 72, Tzarigradsko Chaussee Blvd., 1784 Sofia, Bulgaria

* Correspondence: karekin_esmerian@abv.bg; Tel.: +359-2-979-5811

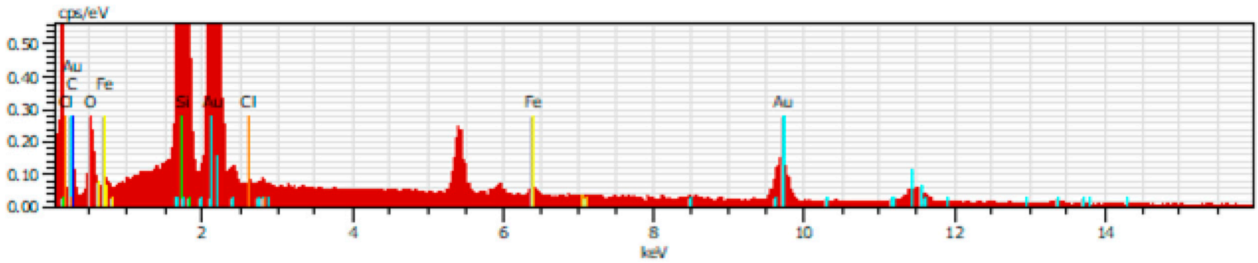

Figure S1. EDX spectrum of the MPF-QCM in the areas without $FeCl_3$ particles.

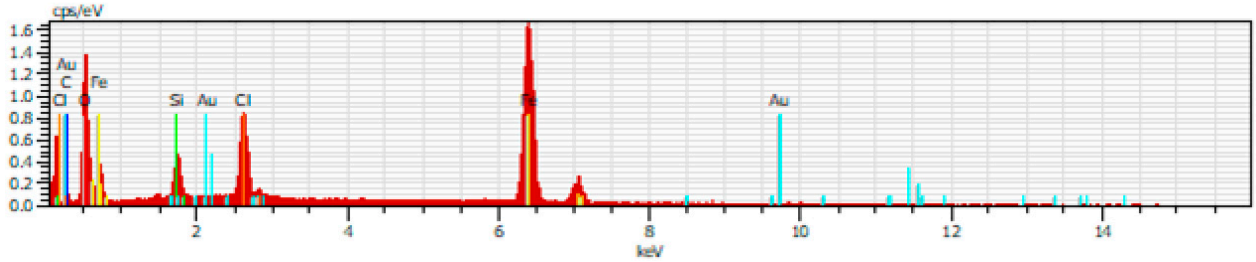

Figure S2. EDX spectrum of the MPF-QCM in the areas with $FeCl_3$ particles.
